# Special Issue of *Cancers*: “Retinoblastoma: Current Challenges and Promising New Approaches”

**DOI:** 10.3390/cancers15082293

**Published:** 2023-04-14

**Authors:** Francis L. Munier

**Affiliations:** Faculté de Médecine et Biologie, University of Lausanne, 1002 Lausanne, Switzerland; francis.munier@unil.ch

Despite being a rare pediatric cancer arising in the developing retina from red/green cone precursors, retinoblastoma is the most common eye cancer worldwide and occupies an emblematic position in oncology and human genetics for the following reasons:

- Historically, the discovery of *RB1* and the recessive nature of its mutations led to the prototypic description of anti-oncogenes or tumor suppressor genes [1].
- Behind its role in retinoblastoma tumorigenesis, the protein encoded by the *RB1*, pRB, belongs to the regulatory INK4A/Cyclin D1/pRB/E2F pathway, universally disrupted in human cancer [2].
- Retinoblastoma is the only pediatric cancer which presents with recognizable signs, allowing awareness campaigns to be credited with significant shortening of the length of time to diagnosis [3]. As such, retinoblastoma was selected as one of the six pediatric cancers which are priorities of the World Health Organization (WHO) Global Initiative for Childhood Cancer (GICC).
- Retinoblastoma is considered as a clinical success story in pediatric oncology, credited with a 5-year survival rate of 99% in high-income countries, giving it the best prognosis among pediatric cancers [4].
- For the first time, the management outcome of heritable retinoblastoma has illustrated the selection relaxation effect of therapeutic intervention for a lethal human disorder via an increased frequency of carriers of germline *RB1* pathogenic variants after only a few generations [5].

As impressive as this may seem, huge efforts must still be made to improve retinoblastoma management. The advent of novel routes of chemotherapy administration, including intra-arterial, intravitreal, and intracameral injections, has already considerably improved the overall eye survival rate, virtually eradicating the need for external beam radiotherapy, eliminating the risk of radio-induced malignancies, and reducing the indications for systemic chemotherapy [4]. These new therapeutic frontiers in the conservative management of RB should be now further extended in order to promote zero tolerance for metastasis, develop new strategies and drugs to overcome multi-drug resistance and toxicity, and improve the visual outcome and quality of life.

This book provides an overview of the current state of the art regarding basic and clinical research as well as the management of retinoblastoma from leading centers in Europe, America, and Asia. 

Among the main topics covered, special emphasis is dedicated to:

- **Retinoblastoma oncogenesis** [6] and **epigenetic regulation** by pRB [7].
- **The differential diagnosis of pediatric intra-ocular tumors and/or pseudo-tumors** (a) in a multicenter prospective observational study of all new diagnoses of pediatric eye tumors over a 5-year period [8], and (b) through the use of magnetic resonance imaging (MRI) features that best differentiate between retinoblastoma and the most common pseudo-retinoblastoma diagnoses [9].
- **Novel genotype–phenotype correlations** by means of a new classification for *RB1* mutations, based on variants’ predicted effect on pRB, notably resulting in the hypothesis that truncated pRB could have a dominant-negative effect on wild-type pRB in a localization-dependent manner [10].
- **The occurrence of second non-ocular benign** [11] **and malignant** [12] **primaries** in two large cohorts of retinoblastoma survivors. The authors showed that 17.6% and 30% of them developed a benign or malignant second primary in the 60 years and 30 years following diagnosis, respectively.
- **The determination of the latest age at which ophthalmological screening under narcosis can be safely discontinued in familial retinoblastoma** [13]. The authors showed that 99% of cases are diagnosed by 3 years and 100% by 4 years of age.
- **The feasibility of proton beam therapy** as a salvage treatment in heavily pre-treated eyes [14] or as a second-choice alternative to combined targeted chemotherapies [15] for eyes with diffuse infiltrating retinoblastoma.
- **Current indications to secondary enucleation** in a consensus paper from the European retinoblastoma group (EURbG), which provided information on when to stop conservative management with absolute and relative criteria for the first time [16].
- **The genomic characterization of metastatic retinoblastoma** in (a) the largest cohort of patients with extra-ocular relapse [17], expanding the list of biomarkers associated with tumors at risk of relapse, and (b) two patients affected by the rare subtype 2 retinoblastoma linked to *MYCN* amplification [18] and metastatic disease to the orbit and lymph nodes resistant to classic systemic chemotherapy regimen. This latter study exemplifies the implementation of customized medicine for retinoblastoma, starting with in vitro cell culture derived from the lymph nodes, followed by high-throughput pharmacological screening to finally identify innovative active drug combinations.
- **The role of liquid biopsies** in the management of retinoblastoma by two leading groups reporting (a) the first series of RB patients to undergo the prospective cfDNA analysis of aqueous humor at the time of diagnosis and longitudinally throughout therapy, setting the basis for larger prospective studies investigating the prognostic value of specific somatic alterations [19]; and (b) a review on plasma and aqueous humor liquid biopsies and their potential to transform the management of retinoblastoma across the clinical spectrum, from prenatal diagnosis and minimally disseminated disease to late malignant sequalae [20].
